# Precision enhancement in wireless capsule endoscopy: a novel transformer-based approach for real-time video object detection

**DOI:** 10.3389/frai.2025.1529814

**Published:** 2025-04-30

**Authors:** Tsedeke Temesgen Habe, Keijo Haataja, Pekka Toivanen

**Affiliations:** School of Computing, University of Eastern Finland, Kuopio, North Savo, Finland

**Keywords:** capsule endoscopy, object detection, real-time processing, transformer models, video analysis, wireless communication, medical imaging, deep learning

## Abstract

**Background:**

Wireless Capsule Endoscopy (WCE) enables non-invasive imaging of the gastrointestinal tract but generates vast video data, making real-time and accurate abnormality detection challenging. Traditional detection methods struggle with uncontrolled illumination, complex textures, and high-speed processing demands.

**Methods:**

This study presents a novel approach using Real-Time Detection Transformer (RT-DETR), a transformer-based object detection model, specifically optimized for WCE video analysis. The model captures contextual information between frames and handles variable image conditions. It was evaluated using the Kvasir-Capsule dataset, with performance assessed across three RT-DETR variants: Small (S), Medium (M), and X-Large (X).

**Results:**

RT-DETR-X achieved the highest detection precision. RT-DETR-M offered a practical trade-off between accuracy and speed, while RT-DETR-S processed frames at 270 FPS, enabling real-time performance. All three models demonstrated improved detection accuracy and computational efficiency compared to baseline methods.

**Discussion:**

The RT-DETR framework significantly enhances precision and real-time performance in gastrointestinal abnormality detection using WCE. Its clinical potential lies in supporting faster and more accurate diagnosis. Future work will focus on further optimization and deployment in endoscopic video analysis systems.

## 1 Introduction

Wireless capsule endoscopy (WCE) is an advanced technique that has been introduced to capture images of the gastrointestinal tract from inside using a capsule that was wireless and could be swallowed by the patient. While in traditional endoscopy, it is only possible to partially examine the small intestine due to the invasive procedure of colonoscopy.

A number of gastrointestinal disorders are frequently seen during wireless capsule endoscopy, such as abnormalities of the ampulla of Vater (Weerakkody et al., 2024), angiectasia (Igawa et al., 2015; Saltzman, 2024), fresh blood and blood hematin (Kimberly and Baillie, 2006), erosion (Feldman et al., 2020), erythema (Ginsberg et al., 2011), foreign bodies (Ikenberry et al., 2011), lymphatic edema (Strober et al., 1967), polyps (Machicado et al., 2020), and ulcers (Kuipers et al., 1995). This study focuses on 10 of these pathology classes for detection and analysis using the Kvasir-Capsule dataset.

However, unlike colonoscopy WCE offers clinical benefits which include early diagnosis of the disease, the technology however poses a challenge in data analysis. WCE procedure can produce more than fifty thousand images, which later creates several hours of video that a specialist has to go through carefully. However, this is not efficient in the sense that it is done manually and involves a lot of time in contrast to the computer-aided ones and this exposes the patient to wrong diagnosis and ability to detect abnormalities that are noticeable. It is therefore important to have automated systems because of the efficiency and accuracy needs of detecting abnormalities in WCE videos in real-time.

Our prior study benchmarked deep learning models for WCE detection, identifying RT-DETR as a promising solution (Habe et al., 2024). The following models were implemented and compared: RT-MDET; RT-MDET variants; SSD; SSD variants; YOLOv3; Faster R-CNN; EfficientDet; and RetinaNet. When these above-mentioned models were used on WCE data, the strength of each model could be seen in terms of the benefits it provided; however, restrictions could also be observed in terms of their weaknesses in the context of the ever-changing environment of the WCE data.

In our earlier studies (Habe et al., 2024), we tested and investigated several deep-learning models to overcome these issues in WCE video analysis. They include RT-MDET variants, SSD variants, YOLOv3, Faster R-CNN, EfficientDet, and RetinaNet, which have been developed and benchmarked. All of these models were useful in terms of the unique features they provided, yet when implemented in the WCE data context, was comprised of certain weaknesses.

- **RT-MDET variants:** These models were intended to work in real-time detection in WCE videos because of their focus on two parameters; efficiency and precision. Although the method was effective in detecting abnormalities that were visualized under stable light conditions and simple structures, they faced issues with low precision with variability in lighting conditions and complex structures.
- **SSD variants and YOLOv3:** These two models are light weight models perfect for real time operation. But at the same time, they have provided significantly worse detection results in most cases, especially in the presence of certain low-contrast abnormalities or areas with poor lighting.
- **Faster R-CNN and EfficientDet:** These models were especially effective in the aspect of detection which was occasionally even higher compared to other techniques in terms of distinguishing the minor elements on the WCE videos. However, this increased their computational complexity and often the processing time and therefore were not as suitable for real time clinical uses.
- **RetinaNet:** This allowed for a more balanced model, which gave good accuracy with realistic processing time. However, like most models, it had its limitations in the fact that it could not easily be applied to WCE data which had different textures and also contained fluids.

### 1.1 Problem statement

The challenges in WCE imaging, particularly in the gastrointestinal tract, are significant. Detecting abnormalities becomes harder because of image quality variability together with visualization blurring and motion artifacts and also mucus and bubbles and food residues that exist in the images (Sadeghi et al., 2024). The model faces difficulties predicting across all pathology types because of its data imbalance. High data volumes (often exceeding 50,000 frames per patient) create complexity in WCE analysis while demanding significant storage capacities along with powerful computational capabilities (Sadeghi et al., 2024; Pascual et al., 2022). The evaluation process by medical experts takes significant time which proves the necessity for advanced automated processing methods. The scarcity of annotated data presents difficulties in training reliable models because additional methods must be employed (Pascual et al., 2022). Algorithmic constraints also play a role, as prior object detection models like Faster R-CNN and YOLOv3 struggle with detecting small lesions and handling the complex, variable textures found in the gastrointestinal tract (Zhang et al., 2024c). Studies have shown that these models often fail due to the intricate background patterns and illumination variability present in WCE datasets (Gui et al., 2024). By implementing real-time processing capabilities and advanced feature extraction methods the RT-DETR model addresses existing limitations in Gastrointestinal endoscopy systems.

### 1.2 Proposed solution

In order to overcome these challenges, we introduce the Real-Time Detection Transformer (RT-DETR) model as a video analysis architecture built with the transformer network. Transformers (Wang et al., 2025) have shown excellent performance in capturing long-range dependencies and, more recently, in computer vision, owing to their capacity to model long distance relations and context in sequences of data. These strengths are utilized in the RT-DETR model to improve the identification of GI pathologies in WCE videos, and particularly in poor light conditions. RT-DETR effectively addresses limitations which are listed in Section 1.1 by leveraging multi-scale feature interaction, an optimized lightweight design, and loss functions, self-attention mechanisms and real-time processing (Zhang et al., 2024a; Lv et al., 2024; Guemas et al., 2024).

### 1.3 Contributions

The primary contributions of this work are as follows:

- **Novel application:** We propose the RT-DETR model for real-time object detection on WCE videos since there are existing models that do not achieve adequate performance.
- **Model enhancements:** Below, we offer several architectural modifications aimed explicitly at enhancing the performance of WCE data, such as the use of various preprocessing techniques and efficient attention mechanisms.
- **Comprehensive evaluation:** We perform several performance evaluation experiments on a highly selected WCE dataset and show that the proposed RT-DETR model is superior to all the other existing models with regard to both accuracy and speed.
- **Clinical relevance:** Thus, the use and implementation of the presented RT-DETR model in diagnosing the WCE can accelerate the process of video analysis and increase the effectiveness of detection results.

## 2 Related work

Various deep learning methods have been applied to WCE diagnosis, evolving from traditional feature-based approaches to advanced deep learning techniques (Bordbar et al., 2023; Varam et al., 2023; Alawode et al., 2024; Alavala et al., 2024; Wu et al., 2023; Oh et al., 2023). These methods aim to enhance detection accuracy and efficiency in analyzing WCE data.

### 2.1 Traditional object detection approaches

Bordbar et al. (2023) conducted a study where a 3D-CNN model is used for multiclass classification of WCE frames which is a major improvement as compared to traditional approaches where handcrafted features are used along with classical machine learning techniques such as SVM. Bordbar et al. (2023) noted that traditional techniques did not do well when handling variability in WCE images and specifically in identifying small and intricate lesions. The 3D-CNN (Bordbar et al., 2023), that included temporal information across frames of the video improved the accuracy but it was still a problem regarding computational load and real-time performance. The implementation of SVM demonstrates exceptional accuracy through its ability to achieve 99.41% detection precision when using color features based on HSI color space to identify between normal and abnormal patterns (Li et al., 2012; Khun et al., 2009). Current research on Random Forest and k-NN methods in WCE detection remains scarce because these classifiers appear in comparative analyses because of their ability to handle diverse datasets while Random Forest offers additional benefits in feature selection and overfitting reduction (Khun et al., 2009). Clinicians face a major challenge with the vast number of WCE images so additional research must focus on creating automated systems which combine deep learning models with current classifiers to boost accuracy and decrease processing times (Varam et al., 2023; Li et al., 2012).

### 2.2 Deep learning in medical imaging

Deep learning techniques have significantly advanced medical imaging applications, particularly in WCE image analysis. Several CNN-based models, including InceptionV3, EfficientNetV2, ResNet, DenseNet, and MobileNet, have been developed to improve feature extraction and classification performance (Varam et al., 2023; Oh et al., 2023). However, CNNs have limitations in incorporating global contextual features, leading to misclassification of visually similar disease classes (Oh et al., 2023). ShuffleNetV1 and ResNet56 demonstrated effectiveness in binary classification tasks but struggled with multiclass cases due to class imbalance and the lack of global contextual information (Wu et al., 2023).

To address these limitations, hybrid architectures have been proposed. The Minimum Spanning Tree (MST) and Spatial Pyramid Pooling, integrated with an EfficientNet-CondConv architecture, have improved hierarchical feature extraction for WCE images (Sharmila and Geetha, 2024). Additionally, PitTree Fusion Algorithms and conditional convolutions have been incorporated to enhance adaptability to varying input resolutions and complexity (Sharmila and Geetha, 2024).

Transformer-based models have also shown promise in WCE analysis. YOLOv8, enhanced with VanillaNet and an Advanced Feature Pyramid Network (AFPN), has been optimized for real-time detection with high feature extraction accuracy (Liang et al., 2024). Comparisons across 14 different CNN-based models indicate that YOLO series models, particularly YOLOv8n, achieve high accuracy and fast inference speeds, reaching up to 416 FPS (Zhang et al., 2024b).

Vision Transformer (ViT) architectures have been introduced to further improve feature extraction. FLATer, a ViT-derived architecture, has demonstrated superior performance by capturing long-range dependencies and global features in endoscopic images (Oh et al., 2023). Swin Transformer and CaiT models, achieving 79.15% accuracy on the Kvasir Capsule dataset and 98.63% on the Red Lesion Endoscopy (RLE) dataset, have been identified as more effective than CNNs due to their ability to model long-distance dependencies (Wu et al., 2023). However, computational overhead remains a limitation in clinical applications.

Recent research has explored Multi-Scale Coupled Attention (MSCA) networks, designed to improve object detection in varying scales (Li et al., 2024a). Ablation studies confirm that MSCCA and MSCSA modules enhance feature recognition precision and stability, making these networks suitable for complex visual scenarios. The combination of CNNs and Transformers has been shown to enhance global feature extraction, leading to improved performance in differentiating GI lesions, including polyps and cancers (Tang et al., 2023).

Furthermore, active learning techniques have been incorporated into ViT models to improve training efficiency in scenarios with limited labeled data (Tang et al., 2023). Despite their advantages, ViTs require extensive computational resources, and further research is needed to optimize these architectures for real-time applications (Li et al., 2024c). Advances in lightweight transformer models and hybrid architectures continue to refine the balance between accuracy, computational efficiency, and real-time diagnostic applicability (Pornvoraphat et al., 2023; Chen et al., 2023).

### 2.3 Real-Time DEtection TRansformer

The VST model that applies T2T-ViT is designed to focus on regions likely to contain polyps, thereby improving detection accuracy (de Moura Lima et al., 2023). The DETR model, when integrated with a ResNet-50 backbone, effectively addresses various object detection tasks, benefiting from transformers' ability to learn long-range dependencies within images (de Moura Lima et al., 2023). These properties enable accurate detection of polyps (de Moura Lima et al., 2023). Additionally, ViT-H/14 has been utilized as the primary classification model for gastroscopic images, leveraging transfer learning with pre-training on the ImageNet-21k dataset (Chae and Cho, 2023). The ViT-H/14 and BiT-L models facilitate relevant feature extraction from small image patches, improving model performance and classification accuracy (Chae and Cho, 2023).

RT-DETR and Deformable DETR models, both based on the Transformer architecture, have been evaluated for real-time object detection (Zhang et al., 2024a). RT-DETR achieves a balanced trade-off between precision and recall, demonstrating high inference speed at 46.9 FPS. Although this is lower than the YOLO series, it remains well-suited for real-time detection tasks (Zhang et al., 2024a).

RT-DETRv2 introduces several enhancements over the original RT-DETR model, optimizing its performance for real-time applications. The deformable attention module has been modified to include multi-scale sampling points, improving the model's ability to learn selective multi-scale features (Lv et al., 2024). Additionally, a discrete sampling operator is employed to replace the grid_sample function, eliminating deployment issues without affecting performance (Lv et al., 2024). Dynamic data augmentation is incorporated, adjusting augmentation strength during training to enhance generalization to target domains (Lv et al., 2024). Moreover, RT-DETRv2 introduces scale-adaptive hyperparameter tuning, which optimizes learning rates based on model size, improving feature quality in smaller networks like ResNet18 while preserving efficiency in larger networks such as ResNet101 (Lv et al., 2024).

A comparative study demonstrated that RT-DETRv2 outperformed its predecessor in both AP and FPS metrics on the COCO dataset across different model sizes (Lv et al., 2024). For instance, RT-DETRv2-S, based on ResNet18, achieved an AP of 47.9, marking a 1.4-point improvement, while maintaining a stable FPS of 217 (Lv et al., 2024). Further ablation studies validated the improvements, showing that reducing sampling points in the deformable attention module did not significantly compromise accuracy while maintaining efficiency (Lv et al., 2024).

Alavala et al. (2024) proposed a pipeline utilizing the Swin Transformer model for classifying WCE frames into bleeding and non-bleeding categories, while RT-DETR was employed for bleeding region detection and segmentation. The Swin Transformer captures both local and global spatial dependencies, while RT-DETR integrates a hybrid encoder and uncertainty-minimal query selection for precise abnormality detection (Alavala et al., 2024). The preprocessing techniques of Lab color space conversion and CLAHE help models perform better through contrast enhancement and artifact reduction according to Alavala et al. (2024). The model attained 66.7% average precision (AP) as well as 98.5% classification accuracy while performing on the validation set (Alavala et al., 2024). The study (Muzammul et al., 2024) introduced a novel approach for UAV aerial image analysis, leveraging Slicing Aided Hyper Inference (SAHI) alongside the RT-DETR-X model. The objective was to improve detection accuracy and efficiency in high-resolution aerial imagery, using the VisDrone-DET dataset for evaluation. The RT-DETR-X model demonstrated real-time object detection capabilities, enhanced by the SAHI method, particularly in identifying small objects within high-resolution scenes (Muzammul et al., 2024).

DETR and Faster R-CNN have also been applied to the localization, detection, and characterization of focal liver lesions (FLLs) in ultrasound images (Dadoun et al., 2022). While DETR achieved superior accuracy with a specificity of 90% and sensitivity of 97%, making it well-suited for real-time clinical applications, Faster R-CNN performed better in certain lesion characterization tasks (Dadoun et al., 2022). This comparison highlights the potential of transformer-based models to enhance diagnostic accuracy in medical imaging.

The RT-DETR model has further been applied for malaria diagnosis by automating the detection and classification of four Plasmodium species in thin blood films (Guemas et al., 2024). The model exhibited high sensitivity, achieving a 90% recall rate in detecting *P. falciparum*. However, distinguishing species such as *P. vivax* and *P. ovale* remains challenging due to their morphological similarities. Overall, RT-DETR was found to be as effective as YOLOv8x for patient-level detection, demonstrating potential for real-time diagnostic applications on low-cost devices, including smartphones (Guemas et al., 2024).

A two-stage detection algorithm incorporating depth maps, Visual Saliency Transformer, and DETR has been developed for polyp detection in colonoscopy images (de Moura Lima et al., 2023). This approach achieved a detection accuracy of 92.6% on the Kvasir-SEG dataset, demonstrating improvements in depth map utilization, saliency extraction, and transformer-based feature learning (de Moura Lima et al., 2023).

The Residual Convolution DETR (RPC-DETR) model introduces several optimizations relevant to medical image analysis, including WCE video detection (Shao et al., 2024). The addition of a Residual Convolution block (RPC-block) enhances feature extraction while reducing computational costs, making it suitable for real-time applications. Additionally, the Shape-IoU loss function improves bounding box regression by accounting for shape variations, which is particularly useful for detecting gastrointestinal abnormalities in WCE images (Shao et al., 2024).

For colorectal cancer screening, YOLOv5 has been enhanced with a P-C3 module and Context Feature Augmentation (CFA) to improve the detection of small and low-contrast polyps in colonoscopy images (Wan et al., 2024b). The integration of a Coordinate Attention Mechanism (CAM) further refines feature selection, enhancing model focus on relevant areas. Evaluation results indicate that the improved YOLOv5 outperformed YOLOv8, RT-DETR R50, and other state-of-the-art methods in polyp detection (Wan et al., 2024b).

The Deformable DETR model has also been applied for breast cancer detection in mammographic images (Xu et al., 2024). The study examined the effectiveness of design choices from Deformable DETR in medical imaging and found that multi-scale feature fusion and complex encoder structures, while beneficial for natural images, may not always improve performance in medical datasets. Instead, simpler architectures were found to be more effective, particularly when handling high-resolution images with small regions of interest (Xu et al., 2024). This insight is relevant to RT-DETR in WCE analysis, as optimizing model complexity may enhance both speed and detection accuracy.

In segmentation tasks, Point SEGTR has been introduced as a deep weakly semi-supervised model derived from DETR (Shi et al., 2023). This framework leverages fully supervised and weakly supervised data, incorporating multi-point and symmetric consistency constraints to improve segmentation stability and effectiveness. Such techniques are particularly beneficial for RT-DETR applications in colonoscopy, where annotated training data is often limited (Shi et al., 2023).

Researchers assessed RT-DETR variants (ResNet18, ResNet34, and ResNet50) when detecting colorectal polyps on both Kvasir-SEG and CVC-ColonDB datasets (Yu et al., 2025). RT-DETR-ResNet34 demonstrated the best AP@0.5 performance with 0.8859 on Kvasir-SEG and 0.8551 on CVC-ColonDB by outscoring RT-DETR-ResNet18 and RT-DETR-ResNet50 in most test cases (Yu et al., 2025). PD-YOLO outperformed all other models in the experiments while demonstrating an AP@0.5 score of 0.8828 on CVC-ColonDB and 0.9478 on Kvasir-SEG and also exhibited better recall values and F1-scores according to the research findings Yu et al. (2025)

### 2.4 Classification of WCE frames

The study classified WCE frames into three categories: Lesion Frames as frames that contain pathologies like ulcers, polyps, and bleeding; Normal Frames as frames with no pathologically altered tissues; and poor frames, in which visibility is compromised due to appearances such as mucus, shadows, or bubbles (Bordbar et al., 2023).

The study was able to categorize the WCE frames into nine classes, and these included foreign body, reduced mucosal view, ileocecal valve, pylorus, ulcer, erosion, lymphangiectasia, erythema, and normal mucosa (Varam et al., 2023). There was high classification accuracy obtained in a variety of gastrointestinal diseases, and therefore the robustness of the ViT (Varam et al., 2023) model in managing diversified diseases was well demonstrated. Thus, some challenges were revealed in the study, the key of which was the Classification issue, especially between apparently similar classes like Erosion and Angiectasia (Varam et al., 2023).

Another author performed the classification of WCE frames based on various categories such as normal frames, inflammatory diseases, vascular lesions, polyps, tumors, and bleeding and achieved a real-time execution with an average frame rate of 30 FPS (Wu et al., 2023).

### 2.5 Datasets

The research utilized a large, publicly available WCE dataset known as the Kvasir-Capsule (Varam et al., 2023; Pogorelov et al., 2017; Smedsrud et al., 2021; Oh et al., 2023; Wu et al., 2023; Sharmila and Geetha, 2024), Red Lesion Endoscopy (RLE) (Wu et al., 2023), Kvasir-SEG (de Moura Lima et al., 2023; Wan et al., 2024b), and ETIS-Larib Polyp DB (Oh et al., 2023; Wan et al., 2024b) datasets. Kvasir-Capsule datasets (Smedsrud et al., 2021) consist of slightly more than 47,238 partially labeled images that were manually reviewed and allocated to one out of 14 categories of gastrointestinal lesions. Because of class imbalance issues, the authors performed under-sampling operations in the preparation of balanced samples for training the models (Varam et al., 2023). This approach was critical in order to prevent class imbalance towards less complex but frequent classes like Normal Mucosa and in enhancing the performance of the model especially with clinically relevant classes which are less frequent (Varam et al., 2023; Oh et al., 2023).

The study (de Moura Lima et al., 2023) uses four public datasets for training and validation: CVC-ClinicDB with 612 images, CVC-ColonDB with 300 images, ETIS-LaribPolypDB with 196 high-resolution images, and Kvasir-SEG with 1,000 images. The study (Chae and Cho, 2023) uses two datasets: Gastroscopic Dataset A and Gastroscopic Dataset B are pathological data of gastric abnormalities and early gastric cancer, and also from AI Hub of the National Information Society Agency of South Korea. This study (Liang et al., 2024) used a dataset from Zhujiang Hospital with 105 GIST pathological slides that was reviewed by two pathologists and adopted data augmentation (Random cropping and Mosaic augmentation).

### 2.6 Summary of related work

The presented literature review also shows the development of object detection and classification models in WCE video analysis from traditional approaches to deep learning. Earlier studies used models such as 3D-CNNs which improved the possibilities to detect spatial and temporal characteristics in the WCE frames but suffered from high computational costs and real-time performance (Bordbar et al., 2023). Progressively with the development of deep learning, models including ResNet (Varam et al., 2023; Oh et al., 2023), EfficientNet (Varam et al., 2023; Oh et al., 2023), and Vision Transformers (ViTs) (Varam et al., 2023; Oh et al., 2023) especially enhance the ability to classify by their potential to capture the global context and the long-range dependence. However, these models can be computationally intensive making their application in real-time clinical settings challenging.

Recent studies have increasingly focused on integrating transformers with CNNs to develop more effective models for handling the ambiguity of WCE data (Sharmila and Geetha, 2024; Liang et al., 2024). Transformer-based architectures have been shown to improve class imbalance issues and enhance the detection of small and intricate lesions (Sharmila and Geetha, 2024; Liang et al., 2024; Lv et al., 2024). Additionally, improvements to models such as RT-DETR have been introduced to enhance real-time object detection, achieving better accuracy while maintaining high operational speed-an essential requirement for clinical applications (Zhang et al., 2024a; Lv et al., 2024; Alavala et al., 2024).

Besides, model innovations, the access to as well as usage of such large and varied data repositories like Kvasir-Capsule and Kvasir-SEG has been critical to the development of such solutions. These datasets together with data augmentation methods have been used to overcome the issues of class imbalance and to enhance the ability of the models to generalize (Varam et al., 2023; Smedsrud et al., 2021; Oh et al., 2023; Wu et al., 2023).

In summary, the related work is consistent with the fact that video analysis in WCE has been progressively enhanced by deep learning and transformer-based models, as well as the ongoing research to improve the accuracy, efficiency, and capacity of handling various medical imaging tasks.

## 3 Methodology

The employed method is designed to enhance the precision, computational effectiveness, and real-time suitability of RT-DETR for WCE video analysis, as suggested in this section. The primary problems in this technique are the class-imbalance problem, architecture improvements, lesion detection accuracy enhancement, and clinical significance of the data. In accordance with best practices, the original developers provided their code, which we used to build the model and benefit from the features and optimizations they introduced (Lv et al., 2024).

### 3.1 Proposed method: RT-DETR with ResNet for WCE pathology detection

In this research, we put forward a customized object detection system that uses the RT-DETR framework, equipped with a modified ResNet backbone for feature extraction. To work with our WCE dataset, we adapt the ResNet architecture by starting with pre-trained weights and fine-tuning the later layers, all while keeping the early layers frozen to preserve valuable features from the pre-training. The Hybrid Encoder exploits multi-scale feature extraction from the varying stages of its backbone to grab both fine and large features essential for finding small pathologies. A customized backbone, integrated with a transformer-based decoder, is designed to enhance both precision and computational performance in WCE video pathology detection.

#### 3.1.1 Data acquisition and preprocessing

The dataset used in this study consists of 16,938 WCE images, covering various gastrointestinal pathologies (Table 1). To ensure compatibility with the RT-DETR model, all images were converted to COCO format for integration with the MMDetection framework. Several preprocessing steps were applied to standardize image dimensions, enhance visual quality, and optimize model performance.

**Table 1:** Performance metrics for RT-DETR small, medium, and large size models as per classes.

**Classes**	**RT-DETR-S**			**RT-DETR-M**			**RT-DETR-X**		
	**Precision**	**Recall**	**F1-Score**	**Precision**	**Recall**	**F1-Score**	**Precision**	**Recall**	**F1-Score**
Ampulla of vater	0.83	0.91	0.87	1.00	1.00	1.00	1.00	1.00	1.00
Angiectasia	0.99	0.99	0.99	1.00	1.00	1.00	1.00	0.99	0.99
Blood fresh	0.98	0.92	0.95	1.00	1.00	1.00	1.00	0.91	0.95
Blood hematin	0.83	0.83	0.83	1.00	1.00	1.00	1.00	1.00	1.00
Erosion	0.97	0.92	0.94	0.98	0.92	0.95	0.97	0.88	0.90
Erythema	0.95	0.95	0.95	0.97	0.97	0.97	1.00	0.95	0.97
Foreign body	1.00	0.97	0.99	0.99	0.99	0.99	0.99	0.99	0.99
Lymphangiectasia	0.97	0.99	0.98	0.99	0.99	0.99	1.00	0.99	0.99
Polyp	1.00	0.93	0.97	1.00	1.00	1.00	1.00	1.00	1.00
Ulcer	0.99	0.97	0.98	0.99	0.97	0.96	0.96	0.94	0.96
Background	0.00	0.00	0.00	0.00	0.00	0.00	0.00	0.00	0.00

Each image was resized to 512 × 512 pixels to maintain uniform input dimensions. EXIF orientation metadata was stripped to ensure consistent pixel alignment. To enhance contrast and improve lesion visibility, contrast adjustment was performed using CLAHE (Contrast Limited Adaptive Histogram Equalization), which enhances local contrast while preventing over-amplification of noise. Normalization was applied using mean and standard deviation scaling to standardize pixel intensity values across the dataset.

To improve generalization and synthetically extend the dataset, data augmentation was applied, generating five additional copies per image. The transformations included:

- Flipping (horizontal and vertical) with a 50% probability.
- Random rotations of 90° (clockwise, counterclockwise, upside-down, or none) and minor random rotations between –12° and +12°.
- Random horizontal shearing between –5° and +5°.
- Random brightness adjustments between –25% and +25%.
- Random exposure corrections between –11% and +11%.
- Gaussian blurring using variable kernel sizes, where the standard deviation for the Gaussian filter was randomly selected between 0 and 3.1 pixels. Since Gaussian filters operate on discrete kernel sizes, the fractional standard deviations were rounded to the nearest applicable kernel size.
- Salt and pepper noise applied to 1.5% of image pixels to simulate real-world noise artifacts.

These augmentations enhance model robustness by ensuring exposure to various transformations that may occur in real-world WCE images.

#### 3.1.2 Data loading strategies

Important steps in the creation and training of models include the loading of data and the techniques used to optimize it. The loading and preprocessing of data is done in parallel which speeds up the training process overall by minimizing the amount of time required to load and prepare each batch and making the best use of the computational resources. After organizing the dataset, additional augmentations were made dynamically during training to increase the effectiveness of model generalization. The augmentations incorporated random photometric distortion with a probability of 50%, random zooming out to provide padding, and random IoU-based cropping with a 80% chance, to teach the model to detect pathologies in different light conditions, scales, and contexts. Bounding boxes were processed to guarantee their validity following transformations. To keep input dimensions consistent, horizontal flipping was randomly applied alongside the resizing of images to 640 × 640 pixels. The augmentation method was applied through epoch 117, at which time augmentations were stopped to stabilize the training process. The batch size of 12 per single GPU was applied for training, along with a validation batch size of 32, which ensured effective data processing in both training and evaluation. However, the developed model uses the weight decay in conjunction with gradient clipping techniques and the AdamW optimizer in conjunction with the dynamic learning rate to improve the training efficiency. All of these techniques provide high accuracy and generality while facilitating quick model convergence.

#### 3.1.3 Model architecture

**Figure 1:**
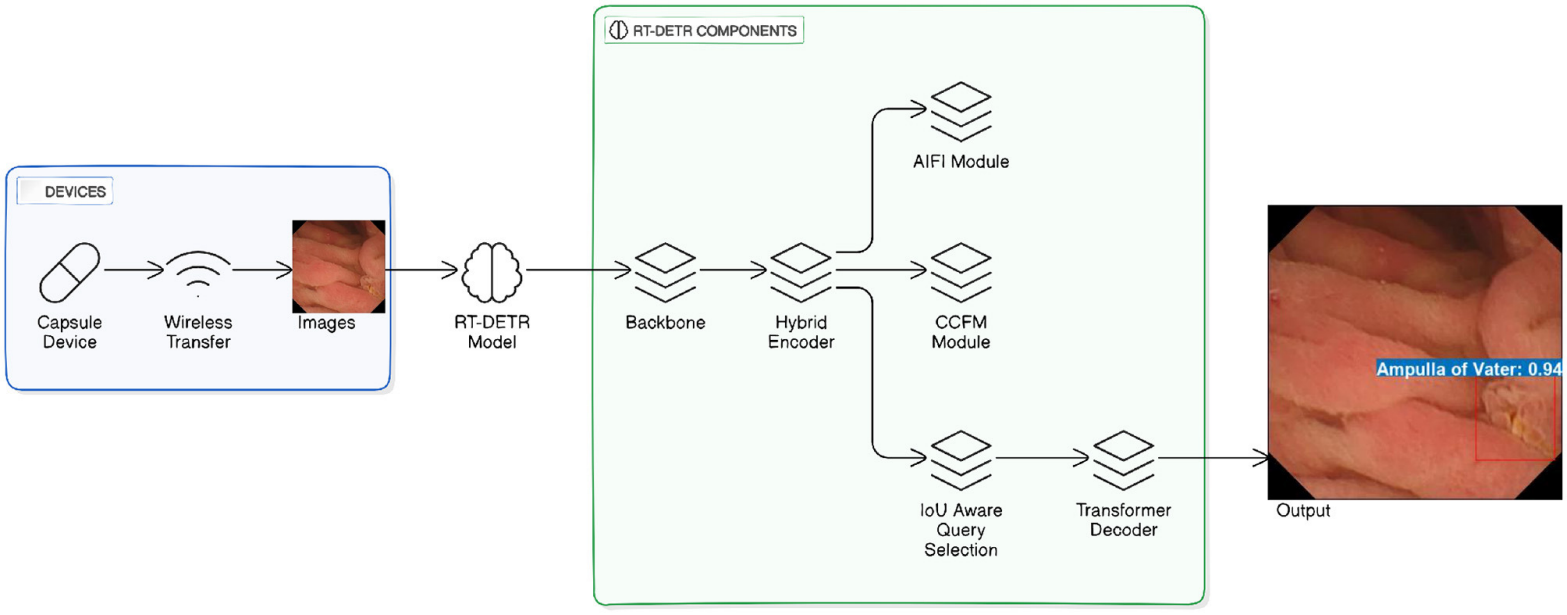
RT-DETR model for wireless capsule endoscopy image detection. The figure is adapted and redrawn by the author based on the architecture presented in Lv et al. (2024).

This work extends the RT-DETR framework, presented by Lv et al. (2024) as shown in Figure 1, aimed at performing real-time object detection. The fundamental part of our model is the different size of ResNet, pre-initialized with ImageNet pre-trained weights. To maintain learned features and modify the model for the WCE dataset, we keep the ResNet lower layers fixed, while fine-tuning the upper layers for pathology detection. Within RT-DETR Model, a backbone (ResNet-18, ResNet-34, or ResNet-101) serves to pull out hierarchical features from the images supplied. These features are then fed into the Hybrid Encoder, which passes the data through several modules: the AIFI Module (Adaptive Intra-Feature Interaction), the CCFM Module (Cross-Scale Context Fusion Module), along with the IoU Aware Query Selection mechanism. The Transformer Decoder carries out final detection and returns predicted bounding boxes and object labels after the IoU Aware Query Selection module enhances object queries prior to their passage (refer Figure 1).

Basically, our architecture integrates efficient feature extraction with transformers' multi-scale abilities to provide accurate and real-time identification of pathologies in WCE data, utilizing pre-trained weights for initialization and fine-tuning the higher layers while leaving the early layers frozen to retain helpful pre-trained features. The Hybrid Encoder takes advantage of multi-scale feature extraction at different backbone stages, which helps it detect both finer and larger features important for the discovery of small pathologies. Integrating this personalized backbone with an effective transformer-based decoder, our approach successfully tackles the problem of detecting pathologies in WCE videos, making sure to provide both precision and timely performance. The RT-DETR model, which is known for its performance in real-time object detection without using NMS has been developed for WCE video analysis problem.

The preference of the RT-DETR model is based on the use of ResNet backbone that is composed of customized connections with the ResNet. The customized ResNet (Lv et al., 2024) connections are beneficial in increasing the gradient flow and decreasing the computation while the residual connections come in handy in the vanishing gradient problem that would allow the training of deeper networks. This backbone is very useful for capturing details in WCE images where many of the features are small and the shapes irregular. The backbone architecture employed is customized structures of varying sizes of ResNet which is to provide both detection accuracy and computational complexity and they include ResNet-18, ResNet-34, and ResNet-101.

RT-DETR incorporates a hybrid encoder that processes WCE images through convolutional layers together with attention mechanisms for local and global feature extraction. The model extracts basic spatial features while concentrating on particular areas of interest. The deformable attention module (Yu et al., 2025; Lv et al., 2024) allows the model to extract features at different resolutions which improves its capability to detect pathology features in lesions with diverse sizes and textures.

Adaptive attention offsets were used for feature selection optimization by altering the receptive field during training. Positional encoding techniques were integrated for the spatial consistency of WCE video sequences. All modifications were implemented following the official RT-DETR repository, ensuring compatibility with transformer-based object detection methods (Zhao et al., 2023; Lv et al., 2024).

### 3.2 Training procedure

The training is carried out step by step to refine the RT-DETR model for WCE pathology detection. Training and validation are carried out on the Kvasir-Capsule dataset, which has been converted to COCO format. The model is trained for 120 epochs with two NVIDIA Quadro RTX 8000 GPUs setting and an overall batch size of 24 (12 per GPU).

A custom loss function is employed, which is a mix of classification, bounding box regression, and localization loss. In order to balance class distributions and enhance detection reliability, the loss components are dynamically weighted. The AdamW optimizer is used with an initial learning rate of 0.0001 under a cosine annealing schedule where the learning rate is progressively decreased. Weight decay of 0.05 and gradient clipping are employed to avoid overfitting and stabilize training.

Scale-adaptive hyperparameters dynamically adjust learning rates based on detector size to ensure consistency in feature extraction across model sizes. Data augmentation techniques outlined in Section 3.1.1 are utilized to increase model robustness and generalization.

### 3.3 Evaluation

Model performance evaluation for WCE pathology detection relies on standard object detection metrics that include accuracy, precision, recall, F1-score, and mean Average Precision together with Intersection over Union. The selected metrics evaluate detection reliability for clinical purposes across various pathology groups. Real-time feasibility is determined by measuring the inference speed (FPS) and performing ROC-AUC analysis as well as confusion matrix evaluations. Robustness testing occurs under different conditions such as illumination levels and image artifacts and frame rates to validate the model's performance. The evaluation procedures follow previously used methods to ensure consistency in result reporting (Habe et al., 2024; Jin and Zhang, 2024; Wu et al., 2023; Li et al., 2024b).

## 4 Results

This study evaluates the performance of three RT-DETR variants RT-DETR-S, RT-DETR-M, and RT-DETR-X on the Kvasir-Capsule dataset for WCE pathology detection. The evaluation considers key metrics such as Average Precision (AP), Recall, F1-score, ROC AUC, and inference speed (FPS). The models are compared against existing object detection methods to assess their accuracy and efficiency.

### 4.1 Model performance and accuracy

The results in Table 2 highlight the high detection accuracy of RT-DETR models. RT-DETR-X achieves the highest AP (78.3%) at IoU 0.50:0.95, confirming its superior capability in pathology detection. However, RT-DETR-M follows closely with AP 78.1%, showing a marginal 0.2 percentage points difference while offering a balanced approach between accuracy and computational efficiency. RT-DETR-S achieves AP 77.8%, demonstrating competitive accuracy while significantly outperforming in inference speed.

**Table 2:** Performance comparison of RT-DETR models.

**Metric**	**RT-DETR-S**	**RT-DETR-M**	**RT-DETR-X**
**Total time(s)**	14.00	17.00	24.00
**Average FPS**	270.52	187.08	59.30
**Evaluation time (s)**	14.00	17.00	24.00
**Average precision (AP) @[IoU=0.50:0.95]**	0.778	0.781	0.783
**AP @[IoU=0.50]**	0.982	0.980	0.974
**AP [IoU=0.75]**	0.841	0.853	0.855
**AP [IoU=0.50:0.95 | area=small]**	0.515	0.471	0.463
**AP [IoU=0.50:0.95 | area=medium]**	0.714	0.720	0.718
**AP [IoU=0.50:0.95 | area=large]**	0.821	0.828	0.836
**Average Recall (AR) [IoU=0.50:0.95 | maxDets=1]**	0.803	0.806	0.807
**AR [IoU=0.50:0.95 | maxDets=10]**	0.820	0.822	0.824
**AR [IoU=0.50:0.95 | maxDets=100]**	0.847	0.854	0.839
**AR [IoU=0.50:0.95 | area=small]**	0.646	0.603	0.556
**AR [IoU=0.50:0.95 | area=medium]**	0.800	0.815	0.792
**AR [IoU=0.50:0.95 | area=large]**	0.884	0.883	0.873

In AP at IoU 0.50, RT-DETR-M scores slightly higher (98.0%) than RT-DETR-X (97.4%), suggesting that it maintains strong detection confidence at a relaxed threshold. However, RT-DETR-X achieves the highest AP at IoU 0.75 (85.5%), making it the most reliable in precise localization of pathology regions. These minor differences indicate that the training process for RT-DETR-X could be further optimized to fully utilize its parameter-rich architecture and maximize performance.

### 4.2 Inference speed and computational efficiency

Inference speed is critical for real-time medical applications. RT-DETR-S achieves the highest FPS (270.52), making it the best choice for real-time WCE analysis. RT-DETR-M follows with 187.08 FPS, offering a strong balance between speed and accuracy. RT-DETR-X, while achieving the highest detection precision, operates at 59.3 FPS due to its larger architecture. Given the Kvasir-Capsule dataset's sizable 47,238 images, RT-DETR-M's competitive performance relative to RT-DETR-X suggests that further tuning of hyperparameters and training epochs for RT-DETR-X might unlock additional gains in accuracy.

These results confirm that RT-DETR models maintain computational efficiency while ensuring high accuracy. RT-DETR-M provides a strong trade-off between inference speed and detection performance, making it ideal for clinical settings where both precision and efficiency are required.

### 4.3 Comparative analysis with other models

A comparison of RT-DETR models with existing object detection frameworks is presented in Table 3. For RT-DETR-M (ours) and RT-DETR-S (ours), Yu et al. (2025), with Kvasir-SEG dataset serves as the direct baseline. RT-DETR-M(ours) achieves an AP50:95 of 78.1%, marking a 7.08 percentage points improvement over RT-DETR-ResNet34 (71.02%) from Yu et al. (2025). Similarly, RT-DETR-S records an AP50:95 of 77.8%, surpassing RT-DETR-ResNet18 (Yu et al., 2025) (70.38%) by 7.42 percentage points. These improvements confirm that our RT-DETR models outperform previous RT-DETR implementations in accuracy while maintaining a better balance between computational efficiency and detection performance.

**Table 3:** Comparative analysis of RT-DETR variants and current object detection models for WCE pathology detection.

**Model**	**Dataset**	**Input Size**	**AP50:95_val_**	**AP50_val_**	**#Params (M)**	**#Epochs**	**FPS**	**Ref**.
YOLOv8-L	WCE-BleedGen	640	68.9	80.2	43	150	-	Alavala et al. (2024)
CRH-YOLO	LDPolypVideo	320	67.8	95.7	0.91	300	96.5	Wan et al. (2024a)
PD-YOLO	CVC-ColonDB	640	70.6	94.7	11.9	300	45.2	Yu et al. (2025)
PD-YOLO	Kvasir-SEG	640	63.9	88.2	11.9	300	45	Yu et al. (2025)
RT-DETR-ResNet50	CVC-ColonDB	640	65.04	84.11	42.8	150	11.8	Yu et al. (2025)
RT-DETR-ResNet18	CVC-ColonDB	640	64.17	85.23	20.1	150	21.9	Yu et al. (2025)
RT-DETR-ResNet34	CVC-ColonDB	640	66.39	85.51	30.1	150	16.9	Yu et al. (2025)
RT-DETR-ResNet50	Kvasir-SEG	640	68.66	88.30	42.8	150	11.3	Yu et al. (2025)
RT-DETR-ResNet34	Kvasir-SEG	640	71.02	88.59	30.1	150	17.3	Yu et al. (2025)
RT-DETR-ResNet18	Kvasir-SEG	640	70.38	89.42	20.1	150	21.6	Yu et al. (2025)
RT-DETR-R101	WCE-BleedGen	640	81.0	66.7	75	150	-	Alavala et al. (2024)
RT-DETR-R50	LDPolypVideo	640	65.2	90.2	42.8	300	17.2	Wan et al. (2024a)
DETR-DC5-R101	WCE-BleedGen	640	72.3	61.2	58	500	-	Alavala et al. (2024)
DETR-R50	WCE-BleedGen	224	73.28	74.47	-	500	-	Alawode et al. (2024)
**RT-DETR-S-R18**	Kvasir-Capsule	640	**77.8**	**98.2**	**20**	**120**	**270.52**	ours
**RT-DETR-M-R34**	Kvasir-Capsule	640	**78.1**	**98.0**	**31**	**120**	**187.08**	ours
**RT-DETR-X-R101**	Kvasir-Capsule	640	**78.3**	**97.4**	**76**	**120**	**59.3**	ours

RT-DETR-S-R18, RT-DETR-M-R34, and RT-DETR-X-R101 denote our models evaluated on the Kvasir-Capsule dataset with different ResNet backbone sizes.

^**^HarDNet-CPS ^**^achieved the highest ^**^AP50 (91.10%)^**^ on Kvasir-SEG, demonstrating strong segmentation performance.

^**^RT-DETR models from PD-YOLO study^**^ (ResNet18, ResNet34, and ResNet50) were tested on ^**^Kvasir-SEG^**^ and ^**^CVC-ColonDB^**^ datasets, showing strong detection accuracy.

^**^RT-DETR-R50 was tested on LDPolypVideo^**^ and showed competitive results with an **AP50 of 90.2% and FPS of 17.2**.

^**^CRH-YOLO achieved the best FPS (96.5) and highest AP50 (95.7%) on LDPolypVideo^**^, showing its efficiency in real-time detection.

^**^Our models achieve the highest AP50 scores, demonstrating their effectiveness for polyp detection in endoscopic images.^**^

RT-DETR achieves an ^**^AP50 of 88.9% on ImageNet-VID^**^ (Chae and Cho, 2023; Hao et al., 2024).

^**^Real-time deep learning processing^**^ enables ^**^WCE video analysis in endoscopic procedures^**^, with an ^**^average inference speed of 14.1 ms^**^ (Sahafi et al., 2022).

The RT-DETR-R101 in Alavala et al. (2024) attains an AP50:95 of 81.0% which is 2.7 percentage points higher than RT-DETR-X (ours). This can be explained by the fact that RT-DETR-R101 (Alavala et al., 2024) has been trained for 150 epochs while RT-DETR-X (ours) for 120 epochs, which has given additional time for feature enhancement. However, RT-DETR-X (ours) reaches a value of 97.4 for AP50 compared to 66.7 for RT-DETR-R101 (Alavala et al., 2024) which shows better detection quality at several IoU thresholds. Because of this, the RT-DETR-R101 (Alavala et al., 2024) has a higher AP50:95 but the AP50 score is lower, it also has the potential to be overfitting, resulting in poor stability in analyzing real-world WCE video.

Comparing RT-DETR models with other object detection frameworks, PD-YOLO (Yu et al., 2025) and CRH-YOLO (Wan et al., 2024a) achieve AP50 scores of 94.7% and 95.7%, respectively. However, their AP50:95 scores drop to 70.6% and 67.8%, indicating weaker localization precision under stricter IoU thresholds. This suggests that while these models excel in high-confidence detections, they struggle with more challenging pathology cases that require refined localization accuracy.

The overall results confirm that RT-DETR-X, RT-DETR-M, and RT-DETR-S demonstrate superior accuracy, robustness, and adaptability for clinical applications. While RT-DETR-R101 (Alavala et al., 2024) reports a slightly higher AP50:95, its lower AP50 score and longer training setup indicate trade-offs in overfitting and computational efficiency. Our models maintain a strong balance between precision, robustness, and feasibility, making them highly suitable for WCE video analysis.

### 4.4 Classification performance and diagnostic precision

The classification results in Table 1 demonstrate that RT-DETR models effectively detect gastrointestinal abnormalities with high accuracy. RT-DETR-M achieves the highest F1-score across most pathology classes, ensuring a balanced trade-off between precision and recall. The model reaches an F1-score of 1.00 for Ampulla of Vater, Angiectasia, Blood Fresh, Blood Hematin, Polyp, and Lymphangiectasia, indicating exceptional reliability in detecting these abnormalities. RT-DETR-X closely follows with comparable performance but records slightly lower recall for Erosion at 0.88 compared to 0.92 for RT-DETR-M, and for Ulcer at 0.94 compared to 0.97. RT-DETR-S maintains competitive classification accuracy, though its recall for Blood Fresh and Erosion remains at 0.92, slightly lower than the other two models. These variations suggest that RT-DETR-M achieves the best balance, while RT-DETR-X offers higher precision for select abnormalities.

**Figure 2:**
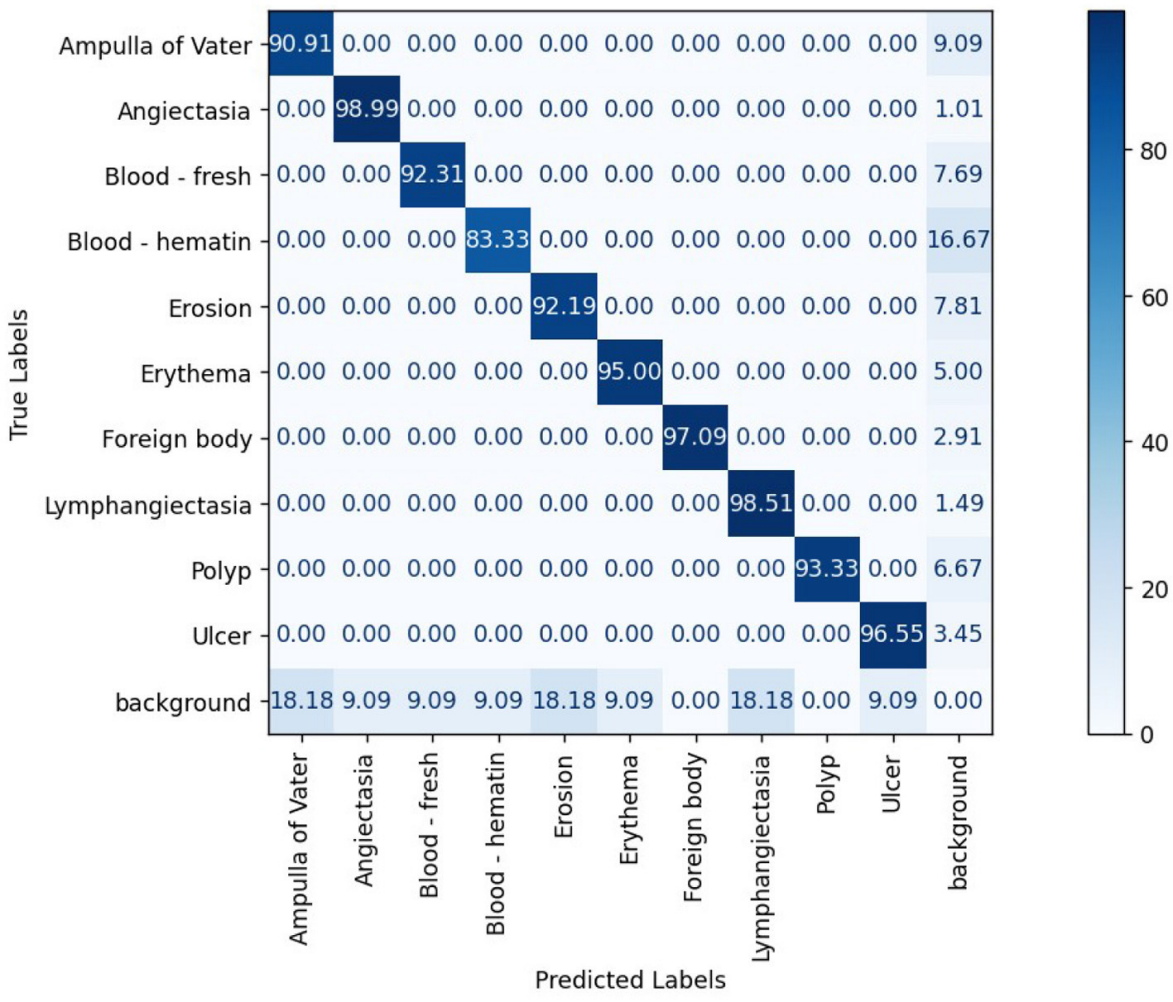
RT-DETR-S normalized confusion matrix.

**Figure 3:**
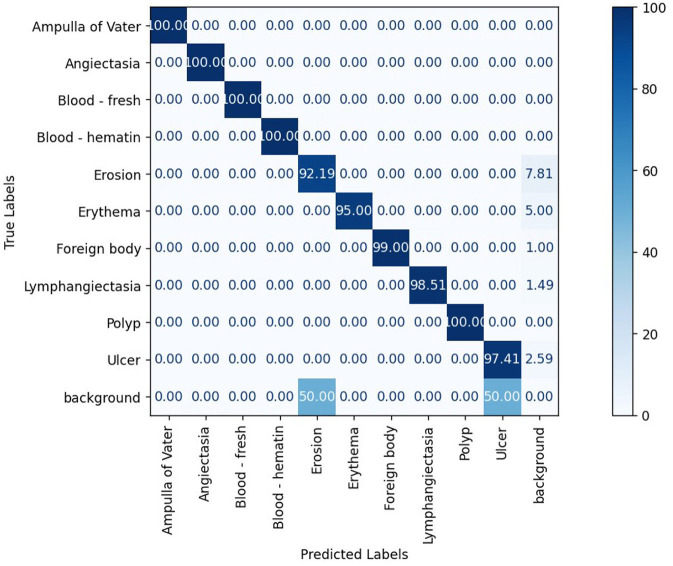
RT-DETR-M normalized confusion matrix.

**Figure 4:**
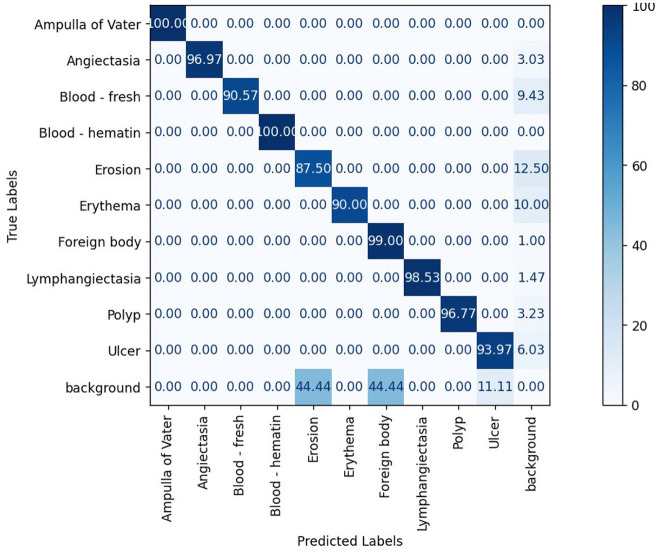
RT-DETR-X normalized confusion matrix.

The confusion matrix analysis in Figures 2–4 further confirms the effectiveness of the models in distinguishing between pathological and non-pathological frames. RT-DETR-M achieves true positive rates exceeding 99% in key pathologies such as Ampulla of Vater, Angiectasia, and Polyp, reinforcing its classification stability. RT-DETR-X performs similarly but shows a slight drop in recall for a few classes. RT-DETR-S, while optimized for real-time performance, still maintains high classification accuracy, though it exhibits a minor reduction in sensitivity for detecting certain abnormalities. The Background class remains consistently undetected across all models, ensuring that the models do not mistakenly classify non-pathological regions as abnormalities.

**Figure 5:**
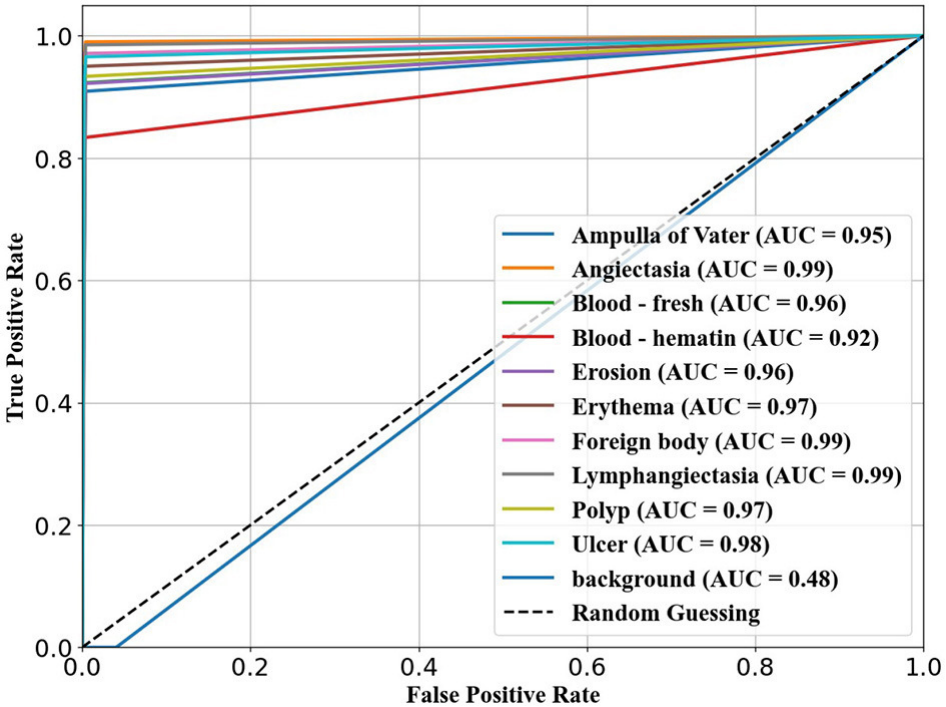
ROC curve for RT-DETR-S.

**Figure 6:**
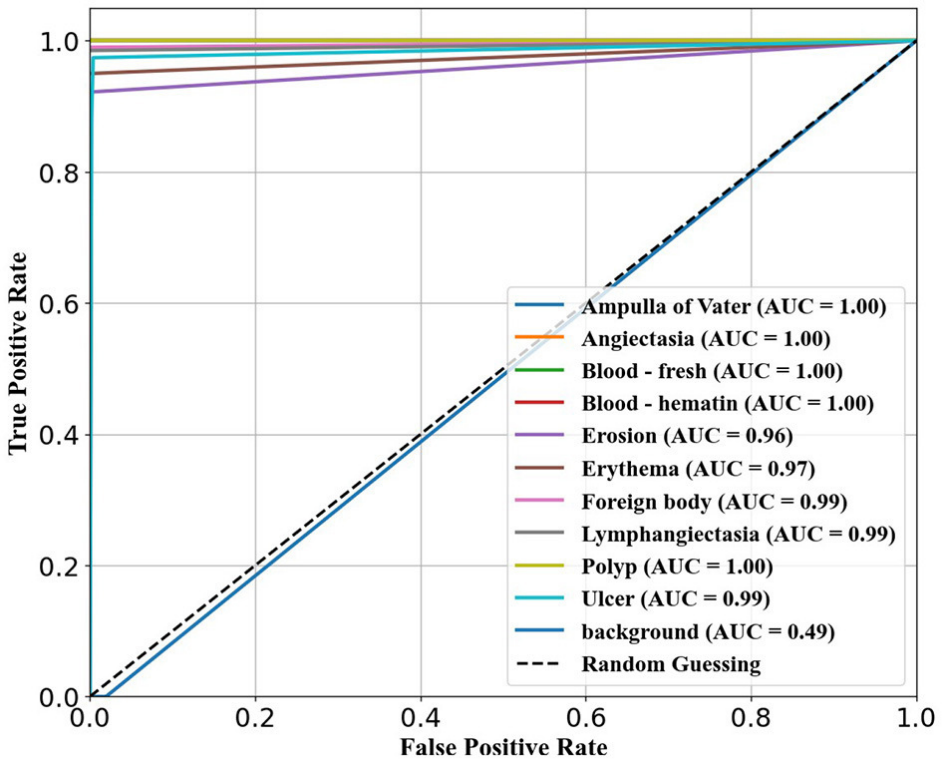
ROC curve for RT-DETR-M.

**Figure 7:**
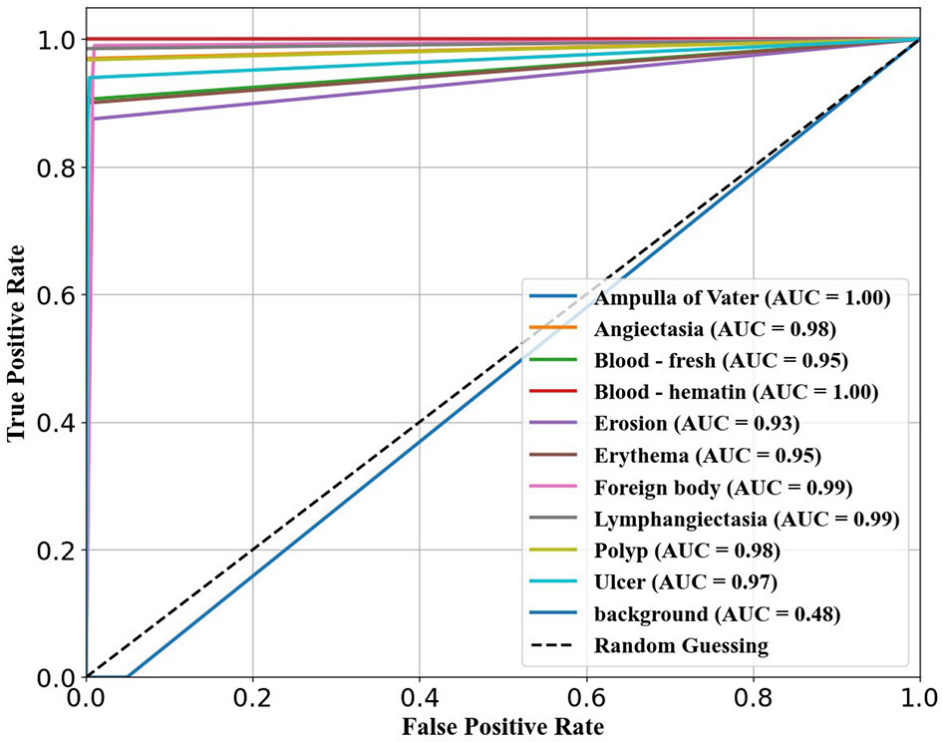
ROC curve for RT-DETR-X.

The ability of the models to differentiate between pathology and non-pathology regions is further supported by the ROC AUC scores in Table 4 and Figures 5–7. RT-DETR-M achieves an ROC AUC of 0.99, confirming its superior ability to generalize across different pathology types. RT-DETR-X follows with an ROC AUC of 0.97, demonstrating high precision in its classifications, while RT-DETR-S, with an ROC AUC of 0.93, remains an efficient model suited for real-time clinical applications.

**Table 4:** Comparative model performance.

**Model variant**	**ROC AUC (Average)**	**Macro Avg**			**Weighted Avg**		
		**Precision**	**Recall**	**F1-Score**	**Precision**	**Recall**	**F1-Score**
RT-DETR-S	0.93	0.87	0.85	0.86	0.96	0.94	0.95
RT-DETR-M	0.99	0.91	0.89	0.90	0.97	0.94	0.95
RT-DETR-X	0.97	0.90	0.87	0.88	0.98	0.97	0.98

## 5 Discussion

The study outcomes demonstrate how different RT-DETR variants affect performance levels when detecting pathologies through WCE. RT-DETR models offer a more adaptable and clinically practical solution for pathology detection in WCE compared to existing object detection frameworks. While models like CRH-YOLO (Wan et al., 2024a) and PD-YOLO (Yu et al., 2025) demonstrate strong performance in specific tasks, they lack the robustness needed for comprehensive WCE analysis, particularly in handling diverse pathology types with varying lesion sizes. The research data shows that higher model complexity does not automatically lead to better accuracy results. The additional parameters in RT-DETR-X lead to only a 0.2 percentage points improvement in AP50:95 detection performance when compared to RT-DETR-M which suggests that model size may not be as important as optimizing training strategies and hyperparameters.

The selection process for models heavily depends on how quickly they can generate inferences. RT-DETR-S offers the fastest frame rate of 270.52 FPS which makes it ideal for real-time diagnostic use. The fast operation of this system leads to slightly diminished detection accuracy for small target objects including ulcers and polyps. The combination of high accuracy and speed performance in RT-DETR-M results in 187.08 FPS making this model the optimal choice for real-time WCE analysis. RT-DETR-X demonstrates the highest AP50:95 score of 78.3 percent but runs at 59.30 FPS which makes it suitable for offline or post-procedure analysis when real-time operation is not necessary.

Model efficiency depends heavily on the backbone architecture design. The RT-DETR-S model with ResNet-18 architecture focuses on speed but struggles to detect smaller or complex abnormalities. The RT-DETR-M network with ResNet-34 architecture demonstrates superior pathology type classification consistency which makes it an optimal selection for medical use. RT-DETR-X utilizes ResNet-101 for feature extraction and detection sensitivity enhancement but requires substantial computational power that hinders its deployment in real-time applications.

The classification performance of RT-DETR-M shows better detection reliability when identifying Fresh Blood and Erosion which ensures reliable medical application detection. The confusion matrix analysis shows that RT-DETR-M delivers an excellent true positive rate which qualifies it as an ideal model for medical applications. The recall performance of RT-DETR-X remains lower than its precision rates which may affect its ability to detect uncommon abnormalities. RT-DETR-S offers enhanced speed performance at the cost of sensitivity which needs thorough examination before clinical implementation.

The ROC AUC analysis demonstrates the reliability of RT-DETR models through its results. RT-DETR-M demonstrates the best performance in AUC value measurements across diverse pathology classes which demonstrates its strong capability to detect abnormalities accurately. RT-DETR-X demonstrates strong performance in medical context since it detects fine lesions though RT-DETR-S stands out due to its speed advantages when operating on WCE videos in real-time conditions.

Researchers should focus their work towards better detection of smaller lesions and enhance model recall efficacy and training techniques to optimize model operational performance. Computational efficiency can be preserved through multi-scale feature extraction techniques and adaptive learning approaches that would improve detection performance. Research should explore how longer training sessions combined with learning rate modifications affect the performance of RT-DETR-X in terms of optimizing its complex structure for better accuracy results.

RT-DETR models establish a flexible method for WCE pathology detection which scales effectively according to different requirements. RT-DETR-S serves real-time diagnostic needs while RT-DETR-M strikes a performance and speed equilibrium and RT-DETR-X provides maximum detection precision for detailed offline evaluations. The flexibility of these models allows for effective integration into clinical workflows, enhancing early disease detection and improving patient outcomes.

## 6 Conclusion

In this work, we addressed the improvement of the accuracy and time efficiency of analyzing WCE video with help of transformer models. The presented methodology employed RT-DETR variants with novel backbones including ResNet-18, ResNet-34, and ResNet-101 as well as HybridEncoder to enhance feature learning. The obtained results in the reformed COCO Kvasir Capsule format with the desired balance between speed and accuracy of the models are presented in above mentioned Table 2 and the highest accuracy for large objects and consistent detection results in our RT-DETR-M and RT-DETR-X as expected. It was found that these improvements in the detection of WCE videos can be linked to the incorporation of object detection transformer models that are particularly useful in detecting long-range features and spatial connections.

The conclusions also underlined the significance of choosing the right backbone; while ResNet-101 showed the best accuracy for important diagnostic tasks, ResNet-34 would be suitable for faster execution without significant loss in precision. Besides, we identified an improvement in HybridEncoder, which is responsible for improving the multi-scale feature extraction, in all the models with regard to detection improvement. This research adds to the current knowledge of transformer models applied to the medical field for analysis of images, and provides a solution with high real-time performance for pathological diagnosis in WCE videos.

**Figure 8:**
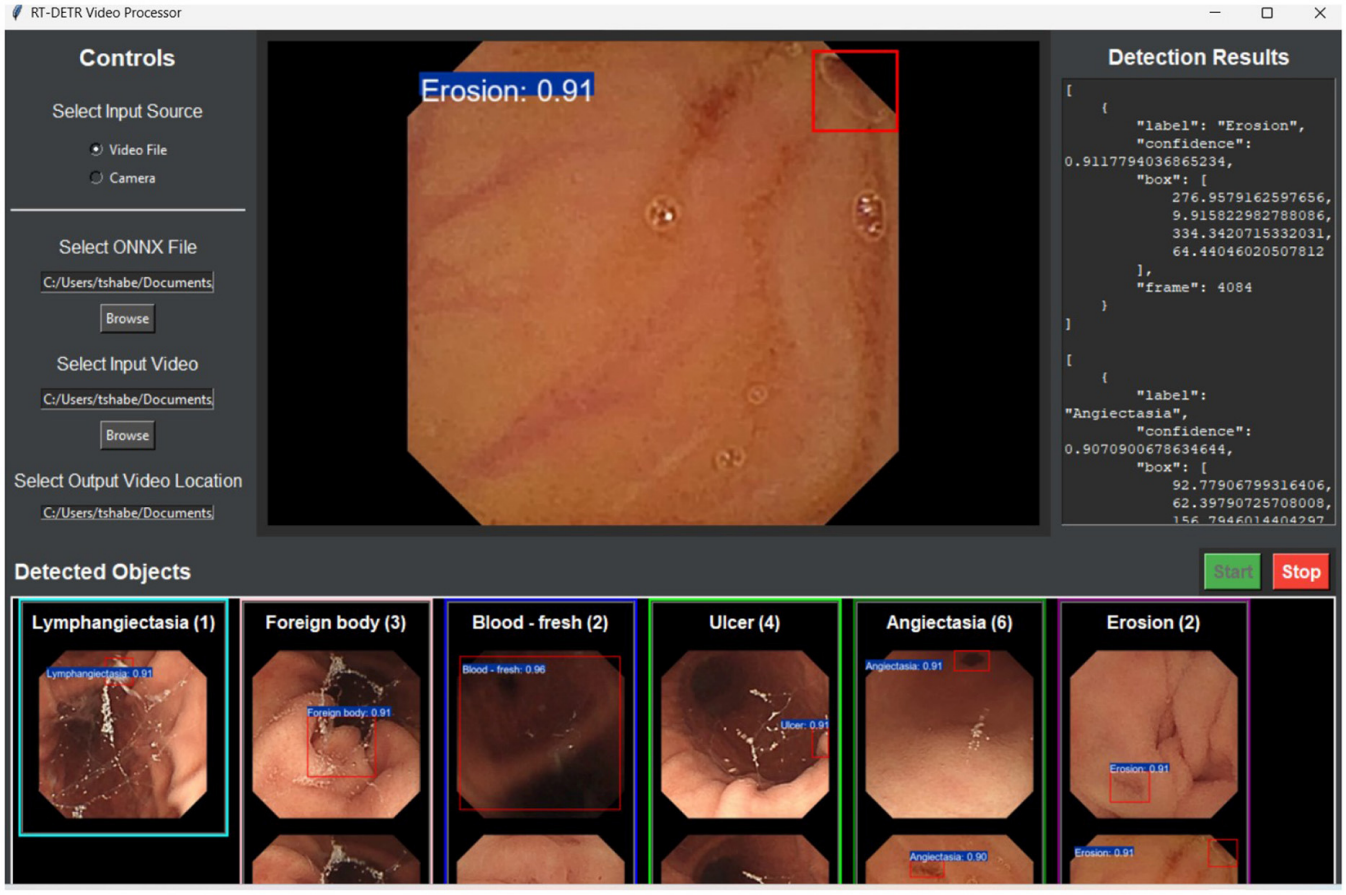
Graphical user interface (GUI) of the RT-DETR video processor.

Future investigations should concentrate on resolving inconsistencies in assessment by fine-tuning hyperparameters, regularization methods, and model structures to enhance the stability and generalizability of larger-size RT-DETR models. To mitigate overfitting, improvements can be made to learning rate schedules, weight decay approaches, and data augmentation techniques, while pruning and lightweight transformer adjustments can boost efficiency. Additionally, validating the models across a diverse range of WCE datasets and integrating real-time optimization methods (such as quantization and hardware acceleration) will ensure that RT-DETR-X maintains reliable accuracy and efficiency in practical applications. The application prototype allowed real-time testing of WCE video analysis for detection assessment as shown in Figure 8.

## Data Availability

Publicly available datasets were analyzed in this study. This data can be found here: https://osf.io/dv2ag/wiki/home/.
